# The Frequency of Sex: Population Genomics Reveals Differences in Recombination and Population Structure of the Aflatoxin-Producing Fungus Aspergillus flavus

**DOI:** 10.1128/mBio.00963-20

**Published:** 2020-07-14

**Authors:** Milton T. Drott, Tatum R. Satterlee, Jeffrey M. Skerker, Brandon T. Pfannenstiel, N. Louise Glass, Nancy P. Keller, Michael G. Milgroom

**Affiliations:** a Department of Medical Microbiology and Immunology, University of Wisconsin—Madison, Madison, Wisconsin, USA; b Department of Genetics, University of Wisconsin—Madison, Madison, Wisconsin, USA; c Innovative Genomics Institute, The University of California, Berkeley, California, USA; d Department of Plant and Microbial Biology, The University of California, Berkeley, California, USA; e Environmental Genomics and Systems Biology, The Lawrence Berkeley National Laboratory, Berkeley, California, USA; f School of Integrative Plant Science, Plant Pathology and Plant-Microbe Biology Section, Cornell University, Ithaca, New York, USA; Cornell University

**Keywords:** *Aspergillus flavus*, sex, recombination, aflatoxin, population structure, population genomics

## Abstract

The apparent rarity of sex in many fungal species has raised questions about how much sex is needed to purge deleterious mutations and how differences in frequency of sex impact fungal evolution. We sought to determine how differences in the extent of recombination between populations of Aspergillus flavus impact the evolution of genes associated with the synthesis of aflatoxin, a notoriously potent carcinogen. We sequenced the genomes of, and quantified aflatoxin production in, 94 isolates of A. flavus sampled from seven states in eastern and central latitudinal transects of the United States. The overall population is subdivided into three genetically differentiated populations (A, B, and C) that differ greatly in their extent of recombination, diversity, and aflatoxin-producing ability. Estimates of the number of recombination events and linkage disequilibrium decay suggest relatively frequent sex only in population A. Population B is sympatric with population A but produces significantly less aflatoxin and is the only population where the inability of nonaflatoxigenic isolates to produce aflatoxin was explained by multiple gene deletions. Population expansion evident in population B suggests a recent introduction or range expansion. Population C is largely nonaflatoxigenic and restricted mainly to northern sampling locations through restricted migration and/or selection. Despite differences in the number and type of mutations in the aflatoxin gene cluster, codon optimization and site frequency differences in synonymous and nonsynonymous mutations suggest that low levels of recombination in some A. flavus populations are sufficient to purge deleterious mutations.

## Abstract (precis)

Differences in the relative frequencies of sexual and asexual reproduction have profound implications for the accumulation of deleterious mutations (Muller’s ratchet), but little is known about how these differences impact the evolution of ecologically important phenotypes. Aspergillus flavus is the main producer of aflatoxin, a notoriously potent carcinogen that often contaminates food. We investigated if differences in the levels of production of aflatoxin by A. flavus could be explained by the accumulation of deleterious mutations due to a lack of recombination. Despite differences in the extent of recombination, variation in aflatoxin production is better explained by the demography and history of specific populations and may suggest important differences in the ecological roles of aflatoxin among populations. Furthermore, the association of aflatoxin production and populations provides a means of predicting the risk of aflatoxin contamination by determining the frequencies of isolates from low- and high-production populations.

## INTRODUCTION

A focus on metazoans has commonized the view that sexual and asexual modes of reproduction are exclusive processes. However, the presence of mixed reproductive modes—in which individual organisms undergo both sexual and asexual reproduction—is common among eukaryotes. Until recently, the large number of fungi thought to be completely asexual stood in marked contrast to our understanding of sex in other eukaryotic lineages. A growing body of population genomic data, however, indicates that many fungal species have retained the ability to reproduce sexually and possess mixed reproductive modes, although the relative importance of sexual and asexual reproduction varies tremendously (1, 2). It is thought that a balance between the costs of sex (3) and the accumulation of deleterious mutations that are inevitable without recombination (4) has shaped the diversity of sexual strategies in fungi (5). Even when present, sex between closely related or identical individuals (e.g., selfing) may not be sufficient to avoid the accumulation of deleterious mutations, especially in haploid species (6). Species with mixed reproductive modes raise fundamental questions about how differences in frequency of sexual reproduction may impact the accumulation of mutations and thus their evolution.

Because of the mix of sexual and asexual reproduction, fungal populations often do not conform to simple population genetic models based on random mating (7). Even rare recombination in mostly asexual populations can give strong signatures of sex, as is evident from fungi that may reproduce sexually only on an annual basis (8). Understanding how much recombination is needed to purge deleterious mutations in these species is further complicated, as the relative frequencies of sexual and clonal reproduction may vary among different populations (9). Populations that differ in sexual frequency are often allopatric (10, 11), reflecting different environmentally related advantages/costs of sex (e.g., recombination load) (12). However, there is evidence that the heterothallic plant-pathogenic ascomycete Aspergillus flavus comprises several genetically isolated sympatric populations in the United States that appear to differ in their relative degrees of recombination and clonality (13, 14).

A. flavus, like many fungi that do not form macroscopic sexual structures, was long thought to be entirely asexual. Population genetic techniques eventually revealed that some recombination has occurred in A. flavus (15, 16), but populations often appear markedly clonal, with specific lineages (often defined by vegetative compatibility groups [VCGs]) frequently being sampled in different fields and years (17). However, high genotypic diversity is suggestive of some recombination between lineages (18, 19). Recently, mating between A. flavus isolates has been achieved in field experiments (20, 21) using extremely high population densities that better reflect artificial biocontrol conditions; i.e., higher densities of nonaflatoxigenic A. flavus propagules have been applied to agricultural fields than would normally exist under agricultural or natural conditions. The idea that A. flavus may be predominantly sexual (21) is difficult to reconcile with a large body of evidence about the predominantly asexual nature of this fungus. Importantly, these field studies occurred on very short timelines and thus do not capture whether the fitness of recombinant progeny is reduced by the disassociation of coadapted traits (i.e., recombination load), and thus sexual reproduction makes little contribution to the overall population. Estimates of sexual frequency from natural populations of A. flavus are complicated by apparent population structure. Failing to realize that populations are genetically subdivided can result in discordance between studies and can mistake genetic differentiation for asexuality within studies (7, 22). In A. flavus, inferences of population structure have used a small number of genetic markers (14) and/or analyzed samples from culture collections, not natural populations (13, 15), and are therefore not sufficient to distinguish inferences of sex from other factors, including selection, demography, and methodology. While some subdivision in the A. flavus population of the United States is evident, the importance of recombination within and between these populations to the extant population structure of this fungus remains unclear.

A. flavus is one of the main producers of aflatoxin, the most potent natural carcinogen known (23–25). An estimated 4.5 billion people are chronically exposed to aflatoxin by consuming contaminated food (26). However, only 40 to 60% of A. flavus isolates produce aflatoxin (27). This polymorphism is maintained through balancing selection (28), which may be mediated by the advantages of aflatoxin production in the presence of insects (29), and the cost of aflatoxin production when the organism competes with soil microbes in their absence (30). Even among aflatoxigenic isolates of A. flavus, levels of aflatoxin production often vary by several orders of magnitude (27). The observation of both a more clonal and a less clonal population of A. flavus (13, 14) raises the possibility that polymorphism for aflatoxin production may be driven in part by differences in the frequency of sex in these populations. In the less sexual population, the accumulation of mutations within the aflatoxin gene cluster that are not purged by recombination may result in decreased aflatoxin production. Indeed, Drott et al. (30) failed to amplify a genetic marker located in the aflatoxin gene cluster from several nonaflatoxigenic isolates, all from the more clonal population. This finding may suggest that previous observation of large deletions in the aflatoxin gene cluster of individual isolates (28, 31) may reflect a lack of recombination of a specific population cluster. We speculate that low aflatoxin producers may be selected against (maladaptive) under conditions favoring aflatoxin production. While quantitative levels of aflatoxin production are generally similar within a clonal lineage, large-scale population surveys of A. flavus in the United States either have not established phylogenetic relationships between isolates (27, 32) or have simply grouped isolates as aflatoxigenic or nonaflatoxigenic rather than examining quantitative differences (14). It is thus unclear if some of the variation in aflatoxin production can be explained by the accumulation of mutations that are not purged by recombination in clonal populations.

The overall objective of this study was to determine if there are differences in the extent of recombination between A. flavus populations in the United States and to investigate whether these differences impact the evolution of the aflatoxin gene cluster, explaining quantitative differences in aflatoxin production. Specifically, we tested the following hypotheses: (i) that population subdivision observed using microsatellite markers are recapitulated and refined by whole-genome sequencing data, (ii) that populations of A. flavus in the United States differ in their extent of recombination, and (iii) that population structure may explain some of the quantitative variation in aflatoxin production previously observed between lineages and that part of these differences can be explained by an accumulation of mutations.

## RESULTS

### Population structure.

All but one of the 95 isolates tested were confirmed as A. flavus, forming a single monophyletic clade with other known A. flavus and Aspergillus oryzae isolates. This clade was distinct from other closely related species (see Table S1 and Fig. S3 in reference 33). The remaining isolate was identified as Aspergillus texensis, a recently described S-type species closely related to A. flavus (34). Measurements of sclerotia (>400 μm in diameter) confirmed that all A. flavus populations comprise large (L-type) isolates (Fig. S4 in reference 33). Isolates in all populations produced far fewer sclerotia than small (S-type) isolates, but those in population B (as defined in the next paragraph) produced significantly more than isolates in either population A or population C (Fig. S4 in reference 33).

**FIG 1:**
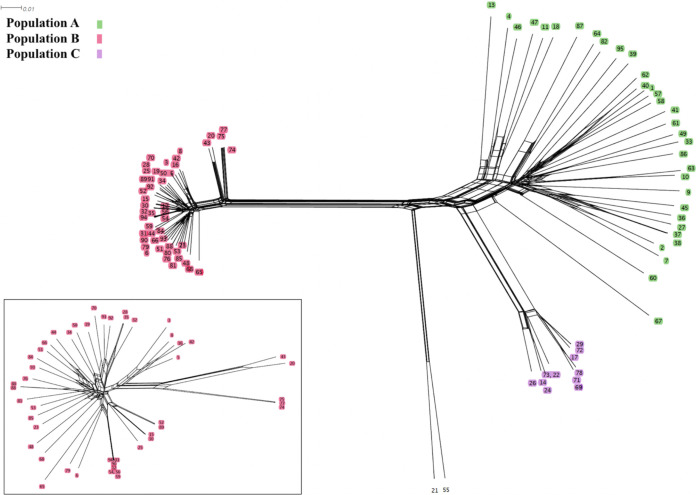
Neighbor-net network of 94 Aspergillus flavus isolates collected in the United States based on 910,777 SNPs. The overall population is subdivided into three populations, A (green), B (red), and C (purple); S-type isolates (two isolates, 21 and 55, at the bottom center of the network) constituted a fourth population but were not colored as they were not included in population-level analyses. Branch tip labels refer to isolates defined in Table S1 (available in reference 33). The network of population B is analyzed separately (shown in the lower left) to demonstrate that loops, which are indicative of recombination, are also present in this population although difficult to visualize in the large graph.

Analysis of the underlying population structure from discriminant analysis of principal components (DAPC) inferred three populations (Fig. 1; Fig. S5 in reference 33). Two of these populations were previously described (populations A and B) using microsatellite markers (14). The third population (referred to here as population C), which is closely related to *A. oryzae* isolates (Fig. S6 in reference 33), was not previously distinguished from population A. Populations B and C have markedly lower diversity than population A (Table 1). All A. flavus isolates sampled in this study were part of what Geiser et al. (15) denoted group I, whereas the *A. texensis* isolate grouped with their group II. Some isolates of populations A and C fell into Geiser et al.’s (15) IB and IC clades. All population B isolates formed a new group from the IA clade, but the use of only three genes did not provide sufficient information to fully resolve populations (Fig. S7 in reference 33). While population structure may also reflect species-level differentiation, *A. texensis*’s inclusion in group II (Fig. S7 in reference 33) and relationships evident in our neighbor-net network (Fig. S6 in reference 33) emphasize that genetic distances to this closely related species are of much larger magnitude than those we find between populations of A. flavus.

**TABLE 1:** Diversity statistics for three populations of Aspergillus flavus in the United States

Population	Correction	No. of tests	*π* ^*a*^	*θ* ^*b*^	Tajima’s *D*^*c*^
A	Uncorrected	33	0.15752	0.002698	–0.0851
	Clone corrected	32	0.15825	0.002882	–0.099
B	Uncorrected	48	0.04071	0.000176	–0.3714
	Clone corrected	37	0.04106	0.000185	–0.4407
C	Uncorrected	11	0.05267	0.000132	0.8958
	Clone corrected	8	0.05566	0.000148	0.8377

a Nucleotide diversity was measured as π, the average number of differences between all possible pairwise comparisons of individuals within a population as defined by Nei and Li (76).

b Population mutation rates are the number of polymorphic sites averaged across the number of sites in the reference genome.

c Tajima’s *D* (59) was calculated in sliding windows of 5,000 bp. Median values are presented as distributions and were not always normal.

We found weak evidence of genetic differentiation based on chemotype only in the full sample of population B, both as isolation by distance (*r *= 0.10, *P = *0.035) and as partitioning of genetic diversity (Ф_PT_ = 0.0365, *P = *0.01) (*P > *0.10 for all other populations). However, these differences were not significant (*P > *0.27) in clone-corrected samples. We interpret this lack of genetic differentiation between chemotypes after clone correction as evidence of recombination between aflatoxigenic and nonaflatoxigenic lineages and/or nonaflatoxigenic isolates arising relatively recently from aflatoxigenic isolates by mutation.

We found evidence of genetic differentiation related to sampling locations only in population A as partitioning of genetic diversity (*Ф*_PT_ = 0.07, *P = *0.01); evidence for isolation by distance was not as strong (*r *= 0.11, *P = *0.078). Clone correction had little impact on either of these effects. While we found no significant genetic differentiation related to sampling location within populations B or C (*P > *0.20), 9 of 11 isolates from population C, including both aflatoxigenic isolates, were found in northern states (Pennsylvania, Indiana, and Iowa). The binomial probability of finding a similar or higher portion of northern isolates in such a sample is 0.0013. Two isolates in population C were sampled in the southern state of Florida, perhaps indicating long-distance migration. We suggest that selection may be a better explanation than restricted migration for the northern distribution of this population. While it has previously been found that the frequency of aflatoxin-producing A. flavus isolates has no association with latitude (14), the presence of a largely nonaflatoxigenic population in the north (population C), where A. flavus population density is remarkably low, raises questions about the potential role of aflatoxin in latitude-associated adaptation.

### Recombination.

We found that all 11 queried meiosis-related genes had conserved protein domains in all populations, suggesting that the ability to recombine sexually is intact. Consistently, mating type allele frequencies were indistinguishable from 0.5 in all three populations (0.46, 0.46, and 0.55 *MAT1-1* for populations A, B, and C, respectively) (Table S1 in reference 33). We performed rarefaction analyses on estimates of recombination and linkage disequilibrium (LD) to validate comparisons between populations that differed in sample size and in total number of single nucleotide polymorphisms (SNPs) (see the supplemental methods in reference 33). Rarefaction analysis using ClonalFrameML found that the median numbers of detectable recombination events were 14,985 for population A and 2,106, and 1,751 for populations B, and C, respectively. In a comparison of the relative contributions of recombination and mutation (*r/m*) to genetic diversity, we found the highest ratios in population C (9.25) and population A (4.09), with a slightly lower value found for population B (2.99) (Table S2 in reference 33). Given the small sample size for population C, we hesitate to interpret this estimate and instead take this as evidence that recombination is an important evolutionary force in all populations, contributing severalfold more diversity than mutation alone.

**FIG 2:**
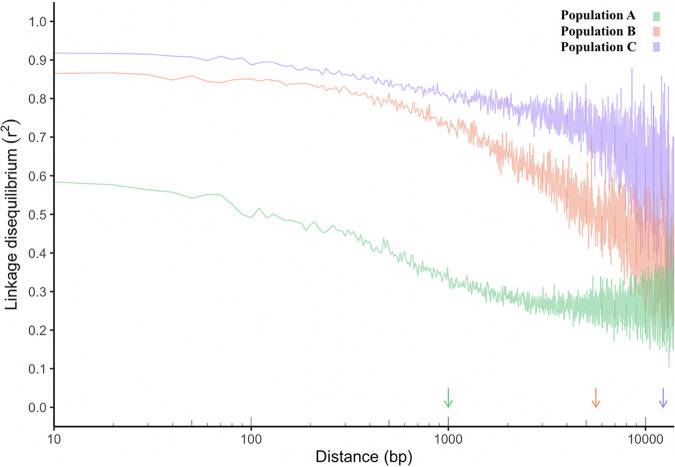
Decay of the linkage disequilibrium (LD) as a function of genomic distance, measured as the correlation between two nucleotides (*r*^2^), from three populations of Aspergillus flavus sampled from the United States, A (green), B (red), and C (purple). To control for differences in sample size and number of SNPs, eight clone-corrected individuals and 50,000 SNPs were randomly sampled 60 times from each population. This plot represents the median decay values. Genomic distances are shown on a log scale. Arrows on the *x* axis indicate the points at which LD was half decayed for each population.

Rarefaction analyses on estimates of LD decay after clone correction found that population A had the shortest median LD decay value (1,000 bp), followed by populations B (5,600) and C (12,300) (Fig. 2). The impact of sample size and number of SNPs on these estimates was evident, as LD decay in full samples (without rarefaction) decreased for all populations, A (200 bp), B (1,300 bp), and C (7,350 bp). These estimates are concordant with ClonalFrameML estimates of the number of recombination events and together constitute evidence of the most recombination in population A, followed by B and then C.

### Analyses of the site frequency spectrum.

We found that all differences in Tajima’s *D* among populations were highly significant (*P < *0.001). While population A had a genome-wide Tajima’s *D* value indicative of neutrality (−0.085), this estimate for population B was slightly negative (−0.44) (Table 2). Negative values of Tajima’s *D* can indicate recovery from a selective sweep (directional selection), recent population expansion, or uneven sampling across unrecognized population subdivisions. In population B, a relative increase (*G *= 163.1, *P < *0.001) in the ratio of nonsynonymous to synonymous evolutionary changes (*dN/dS*) on polymorphic branches (0.269) compared to fixed branches (0.223) for the whole genome is consistent with our interpretation of Tajima’s *D* indicating recent population expansion (Table 2). This same pattern, however, was not found in the aflatoxin gene cluster (*G *= 0.94, *P = *0.332). The magnitude of this finding is likely decreased by our sampling strategy, which was biased against sampling multiple individuals from the same clone.

**TABLE 2:** Comparison of *dN* and *dS* SNP ratios that are either fixed or polymorphic within three populations of Aspergillus flavus in the United States^*a*^

Population	No. of tests	Biallelic SNPs	Fixed		Polymorphic		All	
			Total^*b*^	*dN/dS*	Total	*dN/dS*	Total	*dN/dS*
A	33 (24)	633,259 (1,563)	NA	NA	NA	NA	241,642 (888)	0.265 (0.185)
B	48 (19)	329,900 (1,380)	13,778 (307)	0.223* (0.200)	113,202 (489)	0.269* (0.171)	126,980 (796)	0.263 (0.181)
C	11 (2)	237,719 (980)	5,234 (139)	0.279* (0.321*)	85,018 (375)	0.244* (0.126*)	90,252 (514)	0.246 (0.166)

Total	92 (45)	910,777 (3,295)						

a Ratios of *dN/dS* that are significantly different between fixed and polymorphic data sets are indicated with an asterisk. Results for SNPs in the aflatoxin gene cluster are in parentheses. NA, not applicable. As the number of fixed and polymorphic SNPs was determined pairwise between populations A and B as well as A and C, we did not calculate this value for population A.

b Total number of SNPs in the coding regions only.

In contrast, population C had a positive value for Tajima’s *D* (0.84). A relative decrease in the *dN/dS* of fixed branches (0.279) compared to polymorphic branches (0.244) (*G* = 41.5, *P* < 0.001) (Table 2) was also recapitulated in the aflatoxin gene cluster (*G* = 21.2, *P < *0.001). Coupled with Tajima’s *D* values, these results suggest a recent population bottleneck, perhaps caused by selection, without subsequent population expansion. However, we cannot rule out the possibility that the small sample size for this population may impact results for population C.

We looked for differences in the accumulations of deleterious mutations between populations and found that genome-wide estimates of *dN/dS* ratios were almost identical in A and B (0.265 and 0.263, respectively) and slightly lower in population C (0.246), a difference that may result from the small sample size for this population (*n *= 11) (Table 2). Similarly, while the number of effective codons in highly expressed genes (HEGs) was notably lower than in all other genes, a finding that is consistent with codon optimization, there was no difference in the number of effective codons between populations (Fig. S8 in reference 33). We note that *dN/dS* and codon optimization comparisons are often applied between species and may be of limited value when comparing population-level timescales. Nonetheless, together, these results suggest that the extent of recombination that we observed is sufficient to avoid the accumulation of deleterious mutations on the timescale on which these populations have been diverging.

**FIG 3:**
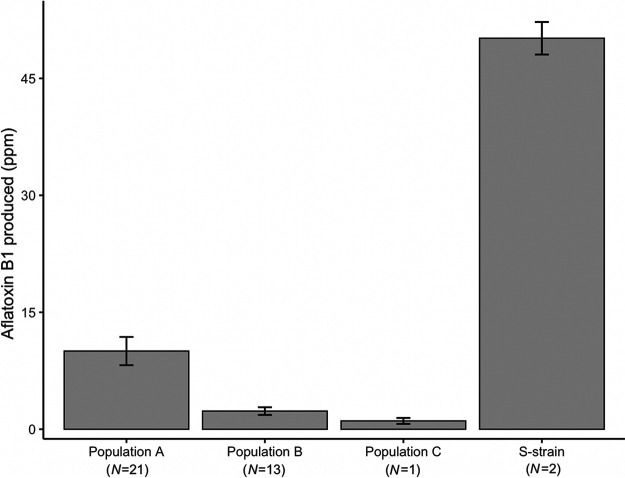
Aflatoxin production of all clone-corrected aflatoxigenic Aspergillus flavus isolates from three populations sampled across the United States, A (*n *= 21), B (*n *= 13), and C (*n *= 1), and from S-strain isolates (*n *= 2). Isolates from population A produced significantly more aflatoxin than those from population B (*P = *0.0038). We did not compare the levels of production of aflatoxin from other groups, as sample sizes were small. Error bars represent the standard errors (SE).

In population B, we observed that of 29 nonaflatoxigenic isolates, 19 were missing the entire aflatoxin gene cluster, whereas another eight were missing the first 12 of 24 genes (*aflT* [FungiDB accession no. AFLA_139420] through *verA* [AFLA_139280]) (Table S1 in reference 33). No genes were found to be deleted from the aflatoxin gene cluster in nonaflatoxigenic isolates from population A or C. Ratios of *dN/dS* in the aflatoxin gene cluster of aflatoxigenic isolates were similar between populations A and B (0.185 [*n *= 24] and 0.181 [*n *= 19], respectively). Because our sample contained only two aflatoxigenic isolates from population C, we did not interpret aflatoxin-related data from this population. We found eight SNPs that impacted stop codons in the aflatoxin gene cluster in population B and six in population A; two were common to both populations. Of these SNPs, five were fixed among aflatoxigenic isolates from population B, indicating that they do not prevent aflatoxin production (several of these occurred very late in the associated proteins), while none of those from population A were fixed. Although no one SNP appears to differentiate the populations, we speculate that the high allele frequencies of many SNPs, with and without high impact, in population B may affect aflatoxin production quantitatively. Consistently with this hypothesis, we find that aflatoxin production was significantly lower in aflatoxigenic isolates from population B than from population A (*P = *0.0038) (Fig. 3).

## DISCUSSION

The A. flavus population in the United States was previously determined to be subdivided into two populations (A and B) using microsatellite markers (14). Here, using whole-genome sequencing, we demonstrate that one of these populations (A) is actually subdivided into two distinct populations (A and C). The divergence of population C from population A appears to be related to geographic distribution, as nine of the 11 isolates in population C were sampled from northern states (Iowa, Indiana, and Pennsylvania) where population densities of A. flavus are typically low (14). Population B, however, appears to have recently undergone population expansion and is distributed widely across the entire sampled range. This result raises the question of why population B has remained genetically isolated from population A, which has a similar geographic distribution. While meiotic machinery appears to be intact, we find less evidence of recombination in populations B and C than in A. However, even these low levels of recombination appear to be sufficient for purging potentially deleterious mutations on the timescale that these populations have been diverging. We suggest that the lack of evidence of recombination between populations A and B could be explained by the rarity of sex in general, and therefore the two populations may have maintained their identities by clonal reproduction.

Recombination is less apparent in populations B and C than in population A, as indicated by both LD decay rates and the number of recombination events inferred. Compared to LD decay rates from a range of fungal species compiled by Nieuwenhuis and James (2), we find that our estimates for population A (after rarefaction) and full-sample estimates for population B are closest to those for a population of Saccharomyces paradoxus (35). This comparison is consistent with previous characterizations of A. flavus population structure (36) because S. paradoxus populations also have clear clonal attributes, with some evidence of recombination. Tsai et al. (11) estimated that a European population of this yeast outcrossed approximately once every 100,000 mitotic generations. Notably, comparison full-sample estimates of LD decay to results of Nieuwenhuis and James (2) suggest that sex in population A is relatively frequent compared to that in many other fungal species. The extent of recombination in population A may have been masked in previous studies by unknowingly pooling individuals from the genetically isolated populations identified here. While such comparisons are useful for understanding the broad context of the sexuality that we observed in A. flavus populations, we urge caution as all measurements of the frequency of sex can be confounded by different sample sizes, selection, a lack of genetic equilibrium, and the demographic histories of sampled populations (37). Indeed, differences observed here between rarified and full data sets emphasize that the ability to detect recombination is affected by the sample size and diversity of populations; comparisons between studies that do not control for these variables must be interpreted cautiously. We note that recent suggestions of frequent recombination in A. flavus may, in part, reflect the biocontrol systems in which they were observed (21), not the entire A. flavus population. Indeed, the AF36 biocontrol strain studied here is derived from the more sexual population, A, and is associated with the most recent evidence of recombination (isolate 1 in Fig. 1; closed loops in this figure are indicative of recombination). However, it is unclear if this finding reflects recent sex with biocontrol strains. The ability to detect recent recombination is related to sampling parents or near parents (38, 39), and thus similar patterns may arise from the intentional introduction of huge numbers of propagules of biocontrol strains in the United States (40), which creates a sampling bias that may increase our ability to detect recombination in this lineage.

Given the relative rarity of recombination in populations B and C, we considered the possibility that these populations may accumulate deleterious mutations, which cannot be purged in the absence of sex. Gioti et al. (6) found that homothallic species of *Neurospora* had elevated *dN/dS* ratios and decreased codon optimization of highly expressed genes, which they interpreted as being a consequence of the failure to purge deleterious mutations in the absence of recombination. However, we did not find similar patterns in populations of A. flavus, regardless of the extent of recombination that we detected. We speculate that large-scale deletions in the aflatoxin gene cluster observed previously (31, 41) may reflect isolates from population B. While these large deletions could result from the accumulation of deleterious mutations and subsequent loss of genes, we find that genome-wide *dN/dS* ratios and evidence of codon optimization from aflatoxigenic isolates in this population are not consistent with the accretion of such mutations. Aflatoxin production by aflatoxigenic isolates was significantly lower in population B than in population A. However, we were unable to establish clear associations between specific SNPs and quantitative differences in aflatoxin production, as SNPs are often linked and/or fixed and unlinked genes are also known to regulate the aflatoxin gene cluster (42). Together we find no clear evidence that low aflatoxin production in population B reflects maladaptation. However, the low mean aflatoxin production of population B raises questions about the adaptive role of low aflatoxin production, although this finding may also reflect a founder affect, not adaptation.

Here, we find that L-type isolates of A. flavus in the eastern and central United States are divided into three distinct populations. While population B produces small amounts of aflatoxin, it is relatively closely related to S-type isolates that produce large amounts (43). Low aflatoxin production in population B may be caused by many fixed alleles in the aflatoxin gene cluster. We do not find evidence that these mutations or that deletions in the aflatoxin cluster are associated with an overall trend in genomic maladaptation from lack of sex. On the timescale that populations have been diverging, it appears that even low levels of sex are sufficient to avoid Muller’s ratchet. Importantly, isolates from population B produce less aflatoxin than isolates from population A. We thus suggest that differentiating the population identities of strains in agricultural fields may be useful for understanding the potential threat of aflatoxin contamination. Our inferences of the number of recombination events and extent of recombination suggest that sex occurs more frequently in population A than in populations B and C. However, these differences may be confounded by population histories, with some populations being more recently derived or introduced and thus not being at equilibrium. Indeed, practically all methods for estimating the frequency of sex require assumptions of long-term equilibrium (37). We speculate that the rarity of sex in population B decreases the number of opportunities for populations to mix and thus may explain how sympatric populations A and B have maintained their identities, although we cannot rule out other possibilities, such as selection or infertility.

## MATERIALS AND METHODS

### Sampling of A. flavus isolates.

A. flavus comprises both large (L-type) and small (S-type) sclerotium-producing isolates. In this paper, we focus on L-type isolates, as they are more common and widely distributed in the United States. We used 93 isolates collected previously (14) from maize field soil in seven states forming both an eastern (Pennsylvania, North Carolina, Florida) and a central (Indiana, Iowa, Oklahoma, and Texas) north-south transect (see Table S1 and Fig. S1 in reference 33). Aflatoxin chemotype was previously determined for all isolates based solely on detection of aflatoxin B_1_ (14). We took a stratified random sample of 90 isolates from 161 L-type isolates that had been genotyped and sorted into two populations using 10 microsatellite markers (14). This sample maximized differences in sampling location, chemotype, and multilocus genotypes (MLGs) and sampled evenly from the two known populations (14). We sampled an MLG only once per state under the assumption that isolates in the same MLG are likely to have nearly identical genomes. We also sequenced three previously uncharacterized S-type isolates (14) and isolates NRRL3357 and AF36 (NRRL18543), which were obtained from the USDA Agricultural Research Service Culture Collection (Peoria, IL).

### DNA extraction and sequencing of A. flavus isolates.

Genomic DNA was extracted from lyophilized tissue of all 95 isolates using phenol-chloroform, as with methods described by Drott et al. (29; supplemental methods in reference 33). Genomic DNA libraries were prepared by Cornell Biotechnology Resource Center Genomics Facility (BRC) using materials and methods equivalent to those used for TruSeq PCR-free library prep (Illumina, San Diego, CA) with eight rounds of PCR. Pooled libraries were sequenced by BRC using an Illumina HiSeq 2500 (Illumina) paired-end 2× 250-bp platform.

### Sequencing and annotation of the NRRL3357 reference genome.

The NRRL3357 genome was resequenced using a combination of PacBio (Pacific Biosciences, Menlo Park, CA) and Nanopore (Nanopore Technologies, Oxford, UK) long reads and Illumina short-read technologies to generate a high-quality reference genome sequence (supplemental methods in reference 33). The final *de novo* assembly consisted of eight gapless scaffolds that represent A. flavus chromosomes with ∼99.99% of bases confirmed. This assembly represents the most complete A. flavus genome assembly generated to date. FGENESH++ 7.2.2 (Softberry Inc., Mount Kisco, NY) was used to reannotate the NRRL3357 genome using a combination of *ab initio* (Fgenesh) and protein homology-based (Fgenesh+) methods.

### Variant calling and genome assembly.

Short reads were quality controlled by using BBMap v38.32 (44), and resulting reads were aligned to the NRRL3357 reference genome using BWA mem v0.7.17 (45). Variants were called using Genome Analysis Toolkit v4.0.12.0 (46) using the best practices pipeline (https://software.broadinstitute.org/gatk/best-practices/workflow?id=11145) while integrating customized hard-filtering parameters for nonmodel organisms. Variants that failed filtration, fell within centromeric regions, or had more than three times the average read depth were removed using VCFtools v1.9 (47).

Genomes were assembled using SPAdes v3.5.0 (48), and resulting assembly qualities were confirmed using QUAST v3.2 (49) and BUSCO v3.0.2 (50). The average assembly had an *N*_50_ of 185 kb, a read depth of 36.5, and contained 98.4% of BUSCO results. Annotation of resulting assemblies was done using Augustus v3.3.2 (51) trained on *A*. *oryzae* gene models.

### Species identification and phenotyping of A. flavus isolates.

We confirmed species-level identification by comparing sequence data from the housekeeping genes *cmdA* (EF661508), *benA* (EF661485), and *RPB2* (EF661440) and the internal transcribed spacer (ITS) ribosomal DNA (rDNA) (AF027863) to sequences from other species in *Aspergillus* section *Flavi* (52). To establish congruence with previous work on A. flavus population structure, we identified the *omtA* (AF036808), *amdS* (AF036768), and *trpC* (AF036851) genes used by Geiser et al. (15) and incorporated existing whole-genome sequence data from five isolates of the closely related Aspergillus oryzae (which is thought to have been domesticated from A. flavus ∼3,000 years ago for use in fermentation, e.g., for soy sauce [53]) from the work of Gibbons et al. (54).

To confirm L-type morphology, we grew two S-type isolates and a subset of 11 randomly selected clone-corrected isolates from each of three identified populations (described below) on both potato dextrose agar (PDA; Difco Laboratories, Detroit, MI) and Czapex-Dox agar (Difco Laboratories) at 30°C. After seven days, the total number of sclerotia was counted, and when possible, 30 randomly selected sclerotia per plate were measured under a dissecting microscope.

To estimate aflatoxin production, we grew 37 clone-corrected aflatoxigenic isolates on PDA at 30°C for 14 days. Aflatoxin was extracted and quantified by high-performance liquid chromatography (HPLC) with UV detection using methods similar to those described previously (55). Differences in aflatoxin production were determined on log-transformed data using a two-tailed *t* test after performing a Shapiro-Wilk test for normality.

### Population structure and population-level metrics.

To confirm previous inferences of population structure (14), we analyzed SNPs using the non-model-based multivariate discriminant analysis of principal components (DAPC) from adegenet v2.1.1 (56) implemented in R v3.5.2 (57) according to procedures outlined in the adegenet tutorial (58). The genetic relationship between individuals was mapped in a neighbor network using SplitsTree v4.14.8 (59). To identify clonal isolates, we calculated genetic distances in poppr v2.8.3 (60).

We determined whether genetic differences between isolates were associated with differences in isolate sampling location (states) and with differences in isolate aflatoxin chemotype using a Mantel test with 1,000 permutations in ade4 v1.7-13 (61) and using an analysis of molecular variance (AMOVA) in poppr (60). Nucleotide diversity, *π*, and population mutation rate, *θ*, were estimated in TASSEL v5.2.51 (62) from a data set of all biallelic SNPs with no missing data. Additionally, Tajima’s *D* (63) was calculated in nonoverlapping sliding windows of 5,000 bp across the entire genome using VCFtools (47). Significant differences in Tajima’s *D* among populations were determined using a Mann-Whitney U test, as done previously (64).

### Evidence of recombination.

We used ClonalFrameML v1.11-3 (65) to detect and quantify the number of recombination events, with phylogenetic relationships generated using RAxML v8.2.1 (66) as described previously (67, 68). Additionally, we interpreted ClonalFrameML estimates of the ratio of the recombination rate to the mutation rate (*R*/θ), the average size of recombination events (δ), the average divergence between donor and recipient (*v*), and the relative importance of recombination and mutation (*r/m*), which is calculated as the product of these three estimates (Table S2 in reference 33). Complementarily, we measured linkage disequilibrium (LD) decay using PLINK v1.90b6.8 (69), as described previously (2). In order to facilitate comparisons between populations with different samples sizes and numbers of SNPs, we performed rarefaction analyses for both ClonalFrameML counts of recombination events (Fig. S2 in reference 33) and LD decay analyses (see the supplemental methods in reference 33).

We determined the mating type of each individual by querying each genome sequence using blastn. Additionally, we assessed whether the protein domains of 11 meiosis-related genes (70) were conserved using the Conserved Domains Tool of the NCBI.

### Analyses of site frequency spectrum.

SNPs were annotated as being synonymous, nonsynonymous, or of high impact (inserting or removing a stop codon) using SnpEff v4.3 (71). Counts of nonsynonymous and synonymous SNPs were converted into *dN* and *dS* using the Nei-Gojobori method (72) with the Jukes-Cantor correction (73). We used the McDonald-Kreitman test to compare the rates of nonsynonymous and synonymous sites that are fixed for different alleles between populations or that are polymorphic in one or both populations. The premise of this test is that *dN/dS* ratios should be similar in fixed sites that manifest in the branches between populations compared to those for polymorphic sites that manifest on branches within a population (74). We used a G test to interpret the significance of *dN/dS* ratios between fixed and polymorphic branches.

To understand the potential effects of reduced recombination in some populations we investigated the possibility that synonymous mutations, while “silent,” may result in a loss of codon optimization. Two data sets from RNA sequencing (RNA-seq) of A. flavus grown at two temperatures (28 and 37°C) were obtained from previous work (75) and used to assess codon optimization across all genes and a subset of highly expressed genes (HEGs), as done previously (Gioti et al. [6]) (see the supplemental methods in reference 33).

### Data availability.

Raw reads for all 95 isolates analyzed here are available in GenBank (BioProject accession no. PRJNA639008). The final scaffolds of the NRRL3357 reference genome have been deposited in GenBank under BioProject accession number PRJNA575750 with associated sequence data available under BioProject accession number PRJNA637788. Gene models for the reference genome are available, as are supplemental figures, tables, and methods in reference 33.

## References

[1] Taylor JW, Hann-Soden C, Branco S, Sylvain I, Ellison CE 2015 Clonal reproduction in fungi. Proc Natl Acad Sci U S A 112:8901–8908. doi:10.1073/pnas.1503159112. 26195774 PMC4517272

[2] Nieuwenhuis BP, James TY 2016 The frequency of sex in fungi. Philos Trans R Soc Lond B Biol Sci 371:20150540. doi:10.1098/rstb.2015.0540. 27619703 PMC5031624

[3] Maynard Smith J 1978 The evolution of sex, vol 4 Cambridge University Press, Cambridge, United Kingdom.

[4] Muller HJ 1932 Some genetic aspects of sex. Am Nat 66:118–138. doi:10.1086/280418.

[5] Billiard S, López‐Villavicencio M, Hood M, Giraud T 2012 Sex, outcrossing and mating types: unsolved questions in fungi and beyond. J Evol Biol 25:1020–1038. doi:10.1111/j.1420-9101.2012.02495.x. 22515640

[6] Gioti A, Stajich JE, Johannesson H 2013 *Neurospora* and the dead‐end hypothesis: genomic consequences of selfing in the model genus. Evolution 67:3600–3616. doi:10.1111/evo.12206. 24299411

[7] Milgroom MG 1996 Recombination and the multilocus structure of fungal populations. Annu Rev Phytopathol 34:457–477. doi:10.1146/annurev.phyto.34.1.457. 15012552

[8] Balloux F, Lehmann L, de Meeûs T 2003 The population genetics of clonal and partially clonal diploids. Genetics 164:1635–1644. 12930767 10.1093/genetics/164.4.1635 PMC1462666

[9] Groth JV, McCain JW, Roelfs AP 1995 Virulence and isozyme diversity of sexual versus asexual collections of *Uromyces appendiculatus* (bean rust fungus). Heredity 75:234–242. doi:10.1038/hdy.1995.131.

[10] Milgroom MG, Sotirovski K, Spica D, Davis JE, Brewer MT, Milev M, Cortesi P 2008 Clonal population structure of the chestnut blight fungus in expanding ranges in southeastern Europe. Mol Ecol 17:4446–4458. doi:10.1111/j.1365-294X.2008.03927.x. 18803594

[11] Tsai IJ, Bensasson D, Burt A, Koufopanou V 2008 Population genomics of the wild yeast *Saccharomyces paradoxus*: quantifying the life cycle. Proc Natl Acad Sci U S A 105:4957–4962. doi:10.1073/pnas.0707314105. 18344325 PMC2290798

[12] Lobkovsky AE, Wolf YI, Koonin EV 2015 Evolvability of an optimal recombination rate. Genome Biol Evol 8:70–77. doi:10.1093/gbe/evv249. 26660159 PMC4758245

[13] Geiser DM, Pitt JI, Taylor JW 1998 Cryptic speciation and recombination in the aflatoxin-producing fungus *Aspergillus flavus*. Proc Natl Acad Sci U S A 95:388–393. doi:10.1073/pnas.95.1.388. 9419385 PMC18233

[14] Drott MT, Fessler LM, Milgroom MG 2019 Population subdivision and the frequency of aflatoxigenic isolates in *Aspergillus flavus* in the United States. Phytopathology 109:878–886. doi:10.1094/PHYTO-07-18-0263-R. 30480472

[15] Geiser DM, Dorner JW, Horn BW, Taylor JW 2000 The phylogenetics of mycotoxin and sclerotium production in *Aspergillus flavus* and *Aspergillus oryzae*. Fungal Genet Biol 31:169–179. doi:10.1006/fgbi.2000.1215. 11273679

[16] Horn BW, Moore GG, Carbone I 2009 Sexual reproduction in *Aspergillus flavus*. Mycologia 101:423–429. doi:10.3852/09-011. 19537215

[17] Mauro A, Battilani P, Callicott KA, Giorni P, Pietri A, Cotty PJ 2013 Structure of an *Aspergillus flavus* population from maize kernels in northern Italy. Int J Food Microbiol 162:1–7. doi:10.1016/j.ijfoodmicro.2012.12.021. 23340386

[18] Horn BW, Greene RL 1995 Vegetative compatibility within populations of *Aspergillus flavus*, *A. parasiticus*, and *A. tamarii* from a peanut field. Mycologia 87:324–332. doi:10.2307/3760829.

[19] Bayman P, Cotty PJ 1991 Vegetative compatibility and genetic diversity in the *Aspergillus flavus* population of a single field. Can J Bot 69:1707–1711. doi:10.1139/b91-216.

[20] Horn BW, Sorensen RB, Lamb MC, Sobolev VS, Olarte RA, Worthington CJ, Carbone I 2014 Sexual reproduction in *Aspergillus flavus* sclerotia naturally produced in corn. Phytopathology 104:75–85. doi:10.1094/PHYTO-05-13-0129-R. 23883157

[21] Lewis M, Carbone I, Luis J, Payne G, Bowen K, Hagan A, Kemerait R, Heiniger R, Ojiambo P 2019 Biocontrol strains differentially shift the genetic structure of indigenous soil populations of *Aspergillus flavus*. Front Microbiol 10:1738. doi:10.3389/fmicb.2019.01738. 31417528 PMC6685141

[22] Taylor J, Jacobson D, Fisher M 1999 The evolution of asexual fungi: reproduction, speciation and classification. Annu Rev Phytopathol 37:197–246. doi:10.1146/annurev.phyto.37.1.197. 11701822

[23] Williams JH, Phillips TD, Jolly PE, Stiles JK, Jolly CM, Aggarwal D 2004 Human aflatoxicosis in developing countries: a review of toxicology, exposure, potential health consequences, and interventions. Am J Clin Nutr 80:1106–1122. doi:10.1093/ajcn/80.5.1106. 15531656

[24] Liu Y, Wu F 2010 Global burden of aflatoxin-induced hepatocellular carcinoma: a risk assessment. Environ Health Perspect 118:818–824. doi:10.1289/ehp.0901388. 20172840 PMC2898859

[25] Wild CP 2007 Aflatoxin exposure in developing countries: the critical interface of agriculture and health. Food Nutr Bull 28:S372–S380. doi:10.1177/15648265070282S217. 17658084

[26] CDC. 2016 Health studies. Understanding chemical exposures. Aflatoxin. CDC, Atlanta, GA http://www.cdc.gov/nceh/hsb/chemicals/aflatoxin.htm. Accessed 2 November 2019.

[27] Horn BW, Dorner JW 1999 Regional differences in production of aflatoxin B_1_ and cyclopiazonic acid by soil isolates of *Aspergillus flavus* along a transect within the United States. Appl Environ Microbiol 65:1444–1449. doi:10.1128/AEM.65.4.1444-1449.1999. 10103234 PMC91204

[28] Moore GG, Singh R, Horn BW, Carbone I 2009 Recombination and lineage-specific gene loss in the aflatoxin gene cluster of *Aspergillus flavus*. Mol Ecol 18:4870–4887. doi:10.1111/j.1365-294X.2009.04414.x. 19895419

[29] Drott MT, Lazzaro BP, Brown DL, Carbone I, Milgroom MG 2017 Balancing selection for aflatoxin in *Aspergillus flavus* is maintained through interference competition with, and fungivory by insects. Proc R Soc B Biol Sci 284:20172408. doi:10.1098/rspb.2017.2408. PMC5745424 29263278

[30] Drott MT, Debenport T, Higgins SA, Buckley DH, Milgroom MG 2019 Fitness cost of aflatoxin production in *Aspergillus flavus* when competing with soil microbes could maintain balancing selection. mBio 10:e02782-18. doi:10.1128/mBio.02782-18. 30782658 PMC6381279

[31] Adhikari BN, Bandyopadhyay R, Cotty PJ 2016 Degeneration of aflatoxin gene clusters in *Aspergillus flavus* from Africa and North America. AMB Express 6:62. doi:10.1186/s13568-016-0228-6. 27576895 PMC5005231

[32] Horn B, Dorner J 1998 Soil populations of *Aspergillu*s species from section *Flavi* along a transect through peanut-growing regions of the United States. Mycologia 90:767–776. doi:10.2307/3761317.

[33] Drott M, Satterlee T, Skerker J, Pfannenstiel BT, Glass NL, Keller NP, Milgroom MG 2020 Supplemental material for “The frequency of sex: population genomics reveals differences in recombination and population structure of the aflatoxin-producing fungus Aspergillus flavus.” figshare doi:10.6084/m9.figshare.12469313. PMC7360929 32665272

[34] Singh P, Orbach MJ, Cotty PJ 2018 *Aspergillus texensis*: a novel aflatoxin producer with S morphology from the United States. Toxins 10:513. doi:10.3390/toxins10120513. PMC6316697 30513994

[35] Bergström A, Simpson JT, Salinas F, Barré B, Parts L, Zia A, Nguyen Ba AN, Moses AM, Louis EJ, Mustonen V, Warringer J, Durbin R, Liti G 2014 A high-definition view of functional genetic variation from natural yeast genomes. Mol Biol Evol 31:872–888. doi:10.1093/molbev/msu037. 24425782 PMC3969562

[36] Horn BW, Ramirez-Prado JH, Carbone I 2009 Sexual reproduction and recombination in the aflatoxin-producing fungus *Aspergillus parasiticus*. Fungal Genet Biol 46:169–175. doi:10.1016/j.fgb.2008.11.004. 19038353

[37] Ennos RA, Hu X-S 2019 Estimating the number of sexual events per generation in a facultatively sexual haploid population. Heredity (Edinb) 122:729–741. doi:10.1038/s41437-018-0171-1. 30531814 PMC6781114

[38] Morton NE 1955 Sequential tests for the detection of linkage. Am J Hum Genet 7:277–318. 13258560 PMC1716611

[39] Kong A, Masson G, Frigge ML, Gylfason A, Zusmanovich P, Thorleifsson G, Olason PI, Ingason A, Steinberg S, Rafnar T, Sulem P, Mouy M, Jonsson F, Thorsteinsdottir U, Gudbjartsson DF, Stefansson H, Stefansson K 2008 Detection of sharing by descent, long-range phasing and haplotype imputation. Nat Genet 40:1068–1075. doi:10.1038/ng.216. 19165921 PMC4540081

[40] Ehrlich K, Moore G, Mellon J, Bhatnagar D 2015 Challenges facing the biological control strategy for eliminating aflatoxin contamination. World Mycotoxin J 8:225–233. doi:10.3920/WMJ2014.1696.

[41] Chang PK, Horn BW, Dorner JW 2005 Sequence breakpoints in the aflatoxin biosynthesis gene cluster and flanking regions in nonaflatoxigenic *Aspergillus flavus* isolates. Fungal Genet Biol 42:914–923. doi:10.1016/j.fgb.2005.07.004. 16154781

[42] Amare MG, Keller NP 2014 Molecular mechanisms of *Aspergillus flavus* secondary metabolism and development. Fungal Genet Biol 66:11–18. doi:10.1016/j.fgb.2014.02.008. 24613992

[43] Bayman P, Cotty PJ 1993 Genetic diversity in *Aspergillus flavus*—association with aflatoxin production and morphology. Can J Bot 71:23–31. doi:10.1139/b93-003.

[44] Bushnell B 2016 BBMap short read aligner. University of California, Berkeley, California http://sourceforge.net/projects/bbmap.

[45] Li H, Durbin R 2009 Fast and accurate short read alignment with Burrows-Wheeler transform. Bioinformatics 25:1754–1760. doi:10.1093/bioinformatics/btp324. 19451168 PMC2705234

[46] McKenna A, Hanna M, Banks E, Sivachenko A, Cibulskis K, Kernytsky A, Garimella K, Altshuler D, Gabriel S, Daly M, DePristo MA 2010 The Genome Analysis Toolkit: a MapReduce framework for analyzing next-generation DNA sequencing data. Genome Res 20:1297–1303. doi:10.1101/gr.107524.110. 20644199 PMC2928508

[47] Danecek P, Auton A, Abecasis G, Albers CA, Banks E, DePristo MA, Handsaker RE, Lunter G, Marth GT, Sherry ST, McVean G, Durbin R, 1000 Genomes Project Analysis Group. 2011 The variant call format and VCFtools. Bioinformatics 27:2156–2158. doi:10.1093/bioinformatics/btr330. 21653522 PMC3137218

[48] Bankevich A, Nurk S, Antipov D, Gurevich AA, Dvorkin M, Kulikov AS, Lesin VM, Nikolenko SI, Pham S, Prjibelski AD, Pyshkin AV, Sirotkin AV, Vyahhi N, Tesler G, Alekseyev MA, Pevzner PA 2012 SPAdes: a new genome assembly algorithm and its applications to single-cell sequencing. J Comput Biol 19:455–477. doi:10.1089/cmb.2012.0021. 22506599 PMC3342519

[49] Gurevich A, Saveliev V, Vyahhi N, Tesler G 2013 QUAST: quality assessment tool for genome assemblies. Bioinformatics 29:1072–1075. doi:10.1093/bioinformatics/btt086. 23422339 PMC3624806

[50] Waterhouse RM, Seppey M, Simão FA, Manni M, Ioannidis P, Klioutchnikov G, Kriventseva EV, Zdobnov EM 2018 BUSCO applications from quality assessments to gene prediction and phylogenomics. Mol Biol Evol 35:543–548. doi:10.1093/molbev/msx319. 29220515 PMC5850278

[51] Stanke M, Morgenstern B 2005 AUGUSTUS: a web server for gene prediction in eukaryotes that allows user-defined constraints. Nucleic Acids Res 33:W465–W467. doi:10.1093/nar/gki458. 15980513 PMC1160219

[52] Frisvad JC, Hubka V, Ezekiel CN, Hong S-B, Nováková A, Chen AJ, Arzanlou M, Larsen TO, Sklenář F, Mahakarnchanakul W, Samson RA, Houbraken J 2019 Taxonomy of *Aspergillus* section Flavi and their production of aflatoxins, ochratoxins and other mycotoxins. Stud Mycol 93:1–63. doi:10.1016/j.simyco.2018.06.001. 30108412 PMC6080641

[53] Machida M, Yamada O, Gomi K 2008 Genomics of *Aspergillus oryzae*: learning from the history of Koji mold and exploration of its future. DNA Res 15:173–183. doi:10.1093/dnares/dsn020. 18820080 PMC2575883

[54] Gibbons JG, Salichos L, Slot JC, Rinker DC, McGary KL, King JG, Klich MA, Tabb DL, McDonald WH, Rokas A 2012 The evolutionary imprint of domestication on genome variation and function of the filamentous fungus *Aspergillus oryzae*. Curr Biol 22:1403–1409. doi:10.1016/j.cub.2012.05.033. 22795693 PMC3416971

[55] Pfannenstiel BT, Greco C, Sukowaty AT, Keller NP 2018 The epigenetic reader SntB regulates secondary metabolism, development and global histone modifications in *Aspergillus flavus*. Fungal Genet Biol 120:9–18. doi:10.1016/j.fgb.2018.08.004. 30130575 PMC6215504

[56] Jombart T 2008 adegenet: a R package for the multivariate analysis of genetic markers. Bioinformatics 24:1403–1405. doi:10.1093/bioinformatics/btn129. 18397895

[57] R Core Team. 2018 R: a language and environment for statistical computing. R Foundation for Statistical Computing, Vienna, Austria.

[58] Jombart T 2008 An introduction to adegenet 2.0.0. Imp Coll London-MRC Cent Outbreak Anal Model, 43.

[59] Huson DH 1998 SplitsTree: analyzing and visualizing evolutionary data. Bioinformatics 14:68–73. doi:10.1093/bioinformatics/14.1.68. 9520503

[60] Kamvar ZN, Tabima JF, Grünwald NJ 2014 Poppr: an R package for genetic analysis of populations with clonal, partially clonal, and/or sexual reproduction. PeerJ 2:e281. doi:10.7717/peerj.281. 24688859 PMC3961149

[61] Dray S, Dufour A-B 2007 The ade4 package: implementing the duality diagram for ecologists. J Stat Soft 22:1–20. doi:10.18637/jss.v022.i04.

[62] Bradbury PJ, Zhang Z, Kroon DE, Casstevens TM, Ramdoss Y, Buckler ES 2007 TASSEL: software for association mapping of complex traits in diverse samples. Bioinformatics 23:2633–2635. doi:10.1093/bioinformatics/btm308. 17586829

[63] Tajima F 1989 Statistical method for testing the neutral mutation hypothesis by DNA polymorphism. Genetics 123:585–595. 2513255 10.1093/genetics/123.3.585 PMC1203831

[64] Cissé OH, Ma L, Wei Huang D, Khil PP, Dekker JP, Kutty G, Bishop L, Liu Y, Deng X, Hauser PM, Pagni M, Hirsch V, Lempicki RA, Stajich JE, Cuomo CA, Kovacs JA 2018 Comparative population genomics analysis of the mammalian fungal pathogen *Pneumocystis*. mBio 9:e00381-18. doi:10.1128/mBio.00381-18. 29739910 PMC5941068

[65] Didelot X, Wilson DJ 2015 ClonalFrameML: efficient inference of recombination in whole bacterial genomes. PLoS Comput Biol 11:e1004041. doi:10.1371/journal.pcbi.1004041. 25675341 PMC4326465

[66] Stamatakis A 2014 RAxML version 8: a tool for phylogenetic analysis and post-analysis of large phylogenies. Bioinformatics 30:1312–1313. doi:10.1093/bioinformatics/btu033. 24451623 PMC3998144

[67] Demené A, Legrand L, Gouzy J, Debuchy R, Saint-Jean G, Fabreguettes O, Dutech C 2019 Whole-genome sequencing reveals recent and frequent genetic recombination between clonal lineages of *Cryphonectria parasitica* in Western Europe. Fungal Genet Biol 130:122–133. doi:10.1016/j.fgb.2019.06.002. 31175938

[68] Stam R, Sghyer H, Tellier A, Hess M, Hückelhoven R 2019 The current epidemic of the barley pathogen *Ramularia collo-cygni* derives from a population expansion and shows global admixture. Phytopathology 109:2161–2168. doi:10.1094/PHYTO-04-19-0117-R. 31322487

[69] Purcell S, Neale B, Todd-Brown K, Thomas L, Ferreira MAR, Bender D, Maller J, Sklar P, de Bakker PIW, Daly MJ, Sham PC 2007 PLINK: a tool set for whole-genome association and population-based linkage analyses. Am J Hum Genet 81:559–575. doi:10.1086/519795. 17701901 PMC1950838

[70] Milgroom MG, Jiménez-Gasco MM, Olivares García C, Drott MT, Jiménez-Díaz RM 2014 Recombination between clonal lineages of the asexual fungus *Verticillium dahliae* detected by genotyping by sequencing. PLoS One 9:e106740. doi:10.1371/journal.pone.0106740. 25181515 PMC4152335

[71] Cingolani P, Platts A, Wang LL, Coon M, Nguyen T, Wang L, Land SJ, Lu X, Ruden DM 2012 A program for annotating and predicting the effects of single nucleotide polymorphisms, SnpEff: SNPs in the genome of *Drosophila melanogaster* strain w1118; iso-2; iso-3. Fly (Austin) 6:80–92. doi:10.4161/fly.19695. 22728672 PMC3679285

[72] Nei M, Gojobori T 1986 Simple methods for estimating the numbers of synonymous and nonsynonymous nucleotide substitutions. Mol Biol Evol 3:418–426. doi:10.1093/oxfordjournals.molbev.a040410. 3444411

[73] Jukes TH, Cantor CR 1969 Evolution of protein molecules. Mamm Protein Metab 3:132.

[74] McDonald JH, Kreitman M 1991 Adaptive protein evolution at the Adh locus in *Drosophila*. Nature 351:652–654. doi:10.1038/351652a0. 1904993

[75] Bai Y, Wang S, Zhong H, Yang Q, Zhang F, Zhuang Z, Yuan J, Nie X, Wang S 2015 Integrative analyses reveal transcriptome-proteome correlation in biological pathways and secondary metabolism clusters in *A. flavus* in response to temperature. Sci Rep 5:14582. doi:10.1038/srep14582. 26416011 PMC4586720

[76] Nei M, Li W-H 1979 Mathematical model for studying genetic variation in terms of restriction endonucleases. Proc Natl Acad Sci U S A 76:5269–5273. doi:10.1073/pnas.76.10.5269. 291943 PMC413122

